# Recent Insights into Plant miRNA Biogenesis: Multiple Layers of miRNA Level Regulation

**DOI:** 10.3390/plants12020342

**Published:** 2023-01-11

**Authors:** Mateusz Bajczyk, Artur Jarmolowski, Monika Jozwiak, Andrzej Pacak, Halina Pietrykowska, Izabela Sierocka, Aleksandra Swida-Barteczka, Lukasz Szewc, Zofia Szweykowska-Kulinska

**Affiliations:** Department of Gene Expression, Institute of Molecular Biology and Biotechnology, Faculty of Biology, Adam Mickiewicz University, Poznań, Uniwersytetu Poznanskiego 6, 61-614 Poznań, Poland

**Keywords:** plants, Arabidopsis, microRNA, miRNA biogenesis, regulation of miRNA level, microprocessor, pri-miRNA degradation, PTGS, core microprocessor evolutionary conservation

## Abstract

MicroRNAs are small RNAs, 20–22 nt long, the main role of which is to downregulate gene expression at the level of mRNAs. MiRNAs are fundamental regulators of plant growth and development in response to internal signals as well as in response to abiotic and biotic factors. Therefore, the deficiency or excess of individual miRNAs is detrimental to particular aspects of a plant’s life. In consequence, the miRNA levels must be appropriately adjusted. To obtain proper expression of each miRNA, their biogenesis is controlled at multiple regulatory layers. Here, we addressed processes discovered to influence miRNA steady-state levels, such as *MIR* transcription, co-transcriptional pri-miRNA processing (including splicing, polyadenylation, microprocessor assembly and activity) and miRNA-encoded peptides synthesis. MiRNA stability, RISC formation and miRNA export out of the nucleus and out of the plant cell also define the levels of miRNAs in various plant tissues. Moreover, we show the evolutionary conservation of miRNA biogenesis core proteins across the plant kingdom.

## 1. Introduction: MicroRNAs as Key Regulators of Plant Life

**Figure 1:**
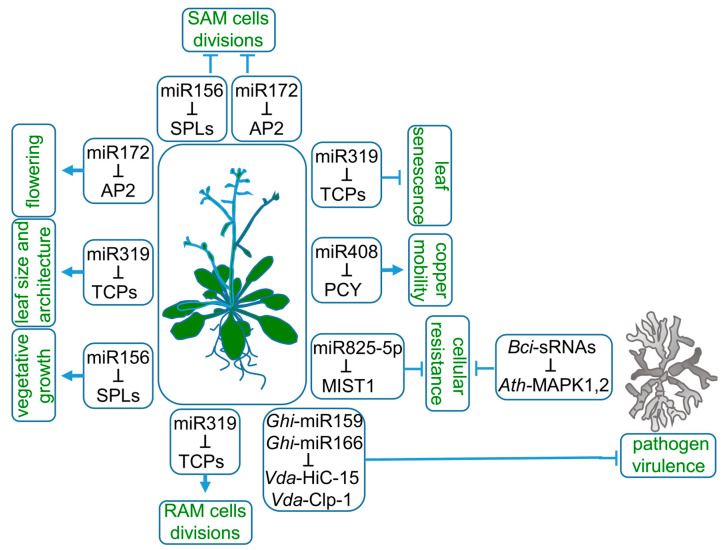
Roles of miRNAs in plant life. MiRNA156, miRNA172 and miRNA319 target SQUAMOSA promoter-binding protein-like (SPLs), APETALA2 (AP2) and TEOSINTE BRANCHED/CYCLOIDEA/PROLIFERATING CELL FACTOR1 (TCPs) mRNAs, respectively, to regulate a wide range of plant characteristics, such as the length of vegetative growth, flowering time, root and leaf architecture. Copper mobility is controlled by miRNA408-targeting mRNA of the copper-sequestrating protein—plantacyanin (PCY). Plant resistance to pathogens is modulated at the cellular level by downregulation of *microrna-silenced toll/interleukin-1 domain* (*MIST1*) transcript by miRNA825-5p. The export of miRNA159 and miRNA166 to pathogen tissues downregulates fungal proteins essential for virulence.

MicroRNAs (miRNAs) represent a class of endogenous, small RNAs, mostly 20–22 nt long. They downregulate gene expression by targeting mRNAs complementary to cleavage or translational inhibition. The biogenesis of miRNAs is completed through the process of miRNA loading on ARGONAUTE (AGO) effector protein to perform cellular tasks. This year marks exactly 30 years since the first report on the existence of miRNA was published. It described lin-4 and let-7 miRNAs present in *Ceanorhabditis elegans* [1]. As for plants, Reinhart et al., 2002, published the first evidence of miRNAs in *Arabidopsis thaliana* [2]. Then, crucial studies were published in a short period of time, not only providing novel plant miRNA families, but also demonstrating the importance of miRNA in plant growth and function [3,4,5,6,7,8]. Examples of miRNAs as essential gene expression regulators are recognized in all aspects of plant life. For instance, their acknowledged function is to coordinate the phasing of the plant life cycle. The transition between vegetative and generative growth is guarded by sequentially expressed miRNA156 and miRNA172 (Figure 1). The length of a juvenile phase of *A. thaliana* life cycle strongly depends on miRNA156 expression. Overexpression of miRNA156 prolongs vegetative growth, while complete depletion of miRNA156 and similar in nucleotide sequence miRNA157 leads to the diminishing of rosette leaves’ formation, which manifests as a flower shoot emerging out of hypocotyl leaves [9,10]. MiRNA156 is also highly expressed in gametophyte-dominated moss *Physcomitrium patens* from the transition from protonema, the juvenile phase of the gametophyte, to adult gamethophores, the leafy shoots of the moss plant [11]. Tobacco side shoots and lateral roots growth are also attributed to miRNA156 [12]. MiRNA156, as well as miRNA157 targets, are members of the SQUAMOSA promoter-binding protein-like (SPL) transcription factors (TFs) family. SPLs are responsible for the induction of early flowering stages and negatively influence the number of juvenile leaves, shoot branching and adventitious roots outgrowth [13]. Therefore, miRNA156 and miRNA157 are recognized as the main drivers of plant vegetative biomass growth.

Entry into the generative phase of plant life is controlled mainly by the switching between miRNA156 and miRNA172 dominance [14,15]. The target of miRNA172, *APETALA2 (AP2)*, encodes a transcription factor belonging to A-class homeotic genes. It has a dual function as a transcriptional activator or repressor of stem cell-maintaining and floral organ identity genes, respectively [16]. During floral primordia development, miRNA172 expression is limited to its center. Therefore, AP2 is expressed in the outer whorls of flower, which then develop into petals or sepals instead of stamens. The expression pattern of miRNA172 and AP2 in developing flower is necessary to release *AGAMOUS (AG)* expression from AP2 repression. In the central part of the flower, AG specifies reproductive organs development [17].

MiRNA172 also accumulates in the shoot meristem, which is needed for the downregulation of AP2-like genes and leads to flowering induction [17]. AP2 is an activator of WUSCHEL, a homeodomain transcription factor maintaining an active stem-cells pool. Therefore, the downregulation of AP2 decreases shoot apical meristem (SAM) activity [18]. The decrease in miRNA156 gradually ends SAM cells’ divisions through a coordinated decline of *AP2*. The upregulated target of miRNA156, SPL15, induces *FRUITFULL* and *MIR172b* genes expression, which are both inhibitors of *AP2* expression [19].

Proper growth of individual plant organs can be differently managed by the same miRNA-target module. In the case of miRNA319, the miRNA-target module is linked to the target multiplicity, redundancy and basal expression pattern variable between organs (Figure 1). MiRNA319 acts in leaf and root morphogenesis by targeting a group of five redundant TEOSINTE BRANCHED/CYCLOIDEA/PROLIFERATING CELL FACTOR1 (TCP) TFs, TCP2, TCP3, TCP4, TCP10 and TCP24 [20]. The expression of miRNA319-regulated TCPs in root under normal conditions is very low [21]. Consequently, knock-out or knock-down mutants of the TCPs do not change root architecture. However, modification of the miRNA319 target site in *TCP4* and its consequent upregulation results in a reduction in cell number in the root apical meristem (RAM) and shortened root length. Therefore, in the root, the miRNA319 role is to eliminate *TCP* transcripts, which allows the proper proliferative activity of RAM and positively influences root length. In leaves, however, the miRNA319-induced downregulation of TCPs correlates not only with leaf size but also with leaf complexity, margins serrations and cotyledon boundaries [20,22,23]. Additionally, miRNA319 deregulation is strongly coupled to senescence-related phenotypes. The miRNA319 target, TCP4 TF, induces the jasmonate biosynthesis enzyme *LIPOXYGENASE2*. Therefore, miRNA319 delays leaf senescence through the decrease in jasmonic acid levels [24].

As there are hundreds of conserved and species-specific miRNAs, their regulatory roles in plants’ adaptation to environmental stimuli are widely studied. MiRNAs are proven to balance plant metabolism during drought, salinity, high or low temperatures, light intensity and nutrient stresses [25,26,27,28]. MiRNAs were proven to control the cellular balance of nitrogen, phosphate, sulfate or copper [29,30,31,32]. Copper intracellular homeostasis is maintained by miRNA408 (Figure 1). The high redox reactivity of copper determines its toxicity; therefore, its overall cellular pool is sequestrated by copper proteins. Moreover, the mobility of copper is strictly regulated. MiRNA408 allocates copper to chloroplasts by targeting mRNA of PLANTACYANIN (PCY), a copper-sequestering protein associated with the cell endomembrane system. A decreased PCY level mobilizes copper, which can be transported to chloroplasts. In chloroplasts, copper plays its biological role as a cofactor of PLASTOCYANIN, an electron-carrier protein that is a part of the photosynthetic electron transport chain. Prolonged darkness induces premature senescence phenotype through PHYTOCHROME-INTERACTING FACTOR3 (PIF3), PIF4 and PIF5 TFs-driven downregulation of *MIR408* expression leads to pooling cooper out of chloroplastic PLASTOCYANIN to endomembrane PCY. This mechanism underlines a major role for miRNA408 in copper remobilization during chloroplast degeneration, which is a part of naturally occurring senescence [33].

Particular metabolic pathways leading to plant resistance against pathogens are also regulated by miRNAs (Figure 1). Brassicaceae-specific miRNA825-5p inhibits basal cellular resistance against *Pseudomonas syringae* by targeting *microrna-silenced toll/interleukin-1 domain* (*TNL1*) (*MIST1*) mRNA to cleavage, which generates phasiRNAs, further enhancing the *MIST1* and other TNL genes’ downregulation [34]. MIST1 belongs to intracellular immune receptors, the role of which is to detect pathogen-derived factors. *MIR825* expression is salicylic acid (SA)-dependent and drops down after a pathogen attack, which releases the *MIST1* from miRNA825-5p control. Therefore, under favorable environmental conditions, miRNA825-5p prevents a family of unnecessary proteins from being translated. Plant resistance includes the export of miRNAs to evolutionarily distinct species, as in the case of cotton and its pathogen *Verticillium dahliae*. Cotton miRNA159 and miRNA166 are exported and act in pathogen tissues [35]. Some pathogens, such as *Botrytis cinerea*, constrain plant AGO1 to utilize Botrytis sRNAs to deregulate AGO1-dependent plant response to extracellular signals [36].

The above examples show how crucial the proper level of a given microRNA is for plant life. The major stage at which microRNA level undergoes regulation is called microRNA biogenesis. In this review, the newest information about detailed mechanisms regulating miRNAs biogenesis are addressed in a stepwise manner. Transcription of *MIRs* and co-transcriptional processes, such as primary miRNA (pri-miRNA) splicing, methylation, as well as stability, influence the abundance of mature miRNA. This is followed by an analysis of the importance of nuclear export and mobility in and out of the source cell. The miRNA biogenesis process is studied mostly in Arabidopsis. Therefore, the evolutionary conservation of basic protein components of microprocessing machinery in plants is also reviewed.

## 2. microRNA Biogenesis—An Overview

**Figure 2:**
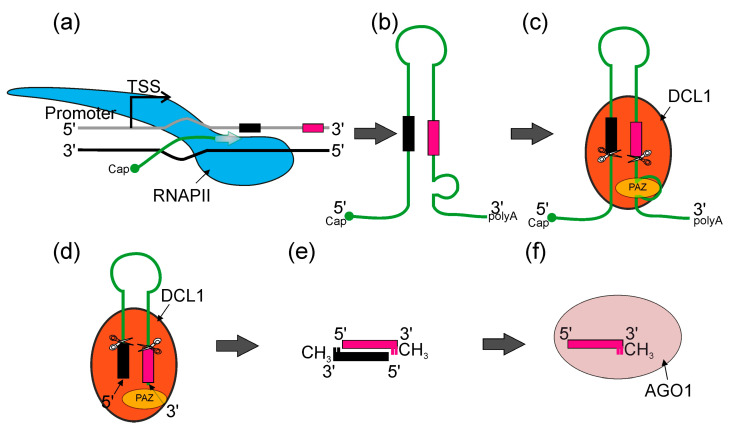
An overview of canonical miRNA biogenesis in plants. (**a**) RNA POLYMERASE II (RNAPII, blue) synthetizes pri-miRNA transcripts which are co-transcriptionally capped and polyadenylated (miRNA*—black bar—and miRNA—magenta bar). (**b**) Pri-miRNA transcripts form stem–loop structures. (**c**) Pri-miRNA transcripts are recognized by DICER-LIKE1 (DCL1) (orange) and other microprocessor proteins (not shown), then pre-miRNAs (stem–loop RNA) are excised with DCL1. DCL1 PAZ domain (yellow) recognizes the internal loop within pri-miRNA and RNase IIIa and RNase IIIb domains (scissors) cut in both strands, leaving two nucleotides overhang on 3′ end of the pre-miRNA. (**d**) Pre-miRNA molecule is translocated towards the PAZ domain and stopped when 3′ overhang reaches the PAZ domain. Then, DCL1 performs the second cut and produces miRNA/miRNA* dsRNA duplexes. (**e**) Later, 3′ ends of each RNA strand undergo methylation. (**f**) ARGONAUTE1 (AGO1) (light pink) binds miRNA and forms together the miRISC (RNA-INDUCED SILENCING COMPLEX).

Plant and animal mechanisms of miRNA action have numerous similarities, such as their critical influence on development and stress responses, followed by the regulation of their target genes. Regarding the biogenesis of these molecules, the main difference is that in animals, the mechanism of biogenesis takes place both in the nucleus and the cytoplasm, while in plants, this process takes place entirely in the cell nucleus [37,38,39,40]. In higher plants, most miRNA genes (*MIR*s) represent independent transcriptional units (intergenic *MIRs*), whereas the remaining part can be found in the introns of protein- or non-coding genes (Figure 2) [41,42]. It was shown that among 167 Arabidopsis pre-miRNA, 97 reside in intergenic regions, 12 in 5′ UTR, 22 in CDS, 23 in introns and 13 in 3′ UTR of other genes [43]. Thus far, it has been shown that all plant *MIR* genes are transcribed by RNA polymerase II (RNA Pol II), which produces primary transcripts (pri-miRNAs) that are 5ʹ capped and 3ʹ polyadenylated. Pri-miRNAs fold into hairpin-like structures that hold miRNA/miRNA* duplexes [44,45,46]. The initial cleavage of the stem–loop structure of the pri-miRNA results in the release of the shorter precursor miRNA (pre-miRNA). Next, the pre-miRNA is processed into miRNA/miRNA* duplexes [46,47,48,49,50] (Figure 2).

Both endonucleolytic cleavage events are catalyzed by the core protein of the microprocessor—DICER-LIKE1 (DCL1), an RNase III type enzyme that recognizes stem–loop structures found in both pri- and pre-miRNAs [47,51,52].

Two other proteins of the microprocessor core—a double-stranded RNA-binding protein HYPONASTIC LEAVES1 (HYL1) and the zinc finger protein SERRATE (SE)—are required for DCL1 to process plant pri-miRNAs efficiently and precisely [46,53,54,55,56,57,58]. Subsequently, the HUA ENHANCER1 methylase (HEN1) methylates the 3’ ends of miRNA/miRNA* duplexes, protecting them from degradation [59,60,61,62]. To date, it is still not fully clear how the miRNA/miRNA* duplexes are integrated into AGO1 and how they are translocated to the cytosol. However, it was shown that matured and methylated miRNAs are loaded into AGO1 in the nucleus and exported to the cytosol as AGO1: miRNA complexes (RNA-induced silencing complex, miRISC) via chromosomal maintenance1/exportin1 (CRM1/EXPO1) [63,64]. The miRNA* is then eliminated, leaving only the miRNA in the miRISC complex [65,66,67,68,69]. While the basic model of miRNA biogenesis appears to be fully described, there are still many insufficiently understood details in it. Understanding these is necessary to compose a coherent and comprehensive picture of the miRNA biogenesis phenomenon. Therefore, in this review, we discuss the latest reports on the hitherto unanswered issues in this complex process.

## 3. microRNA Transcription

**Figure 3:**
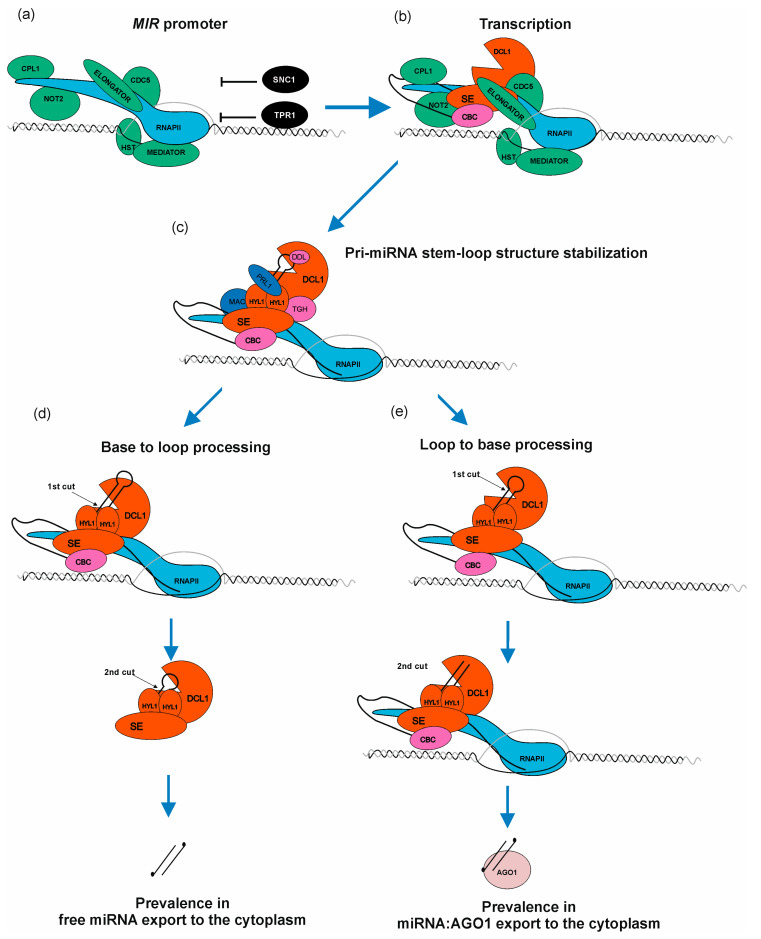
MicroRNA biogenesis is a complex process requiring a plethora of accessory proteins to regulate a proper level of mature miRNA. (**a**) RNAPII, ELONGATOR complex, MEDIATOR complex, C-TERMINAL DOMAIN PHOSPHATASE-LIKE1 protein (CPL1), NEGATIVE ON TATA LESS2 protein (NOT2), CELL DIVISION CYCLE5 (CDC5), HASTY (HST), SUPPRESSOR OF NPR1-1, CONSTITUTIVE1 (SNC1) as well as TOPLESS-RELATED PROTEIN1 (TPR1) bind to *MIR* loci. Green color marks positive regulators of miRNA transcription and black color marks negative regulators. (**b**) miRNA gene is transcribed. Nuclear CAP-BINDING PROTEIN COMPLEX (CBC) binds to newly synthesized cap structure; SERRATE (SE) and DICER LIKE1 protein (DCL1) are assembled to miRNA precursor. (**c**) Pri-miRNA stem–loop structure is bound by HYPONASTIC LEAVES1 (HYL1) to stabilize the miRNA precursor hairpin. TOUGH (TGH), PLEIOTROPIC REGULATORY LOCUS1 (PRL1), MOS4-ASSOCIATED COMPLEX (MAC) and DAWDLE (DDL) assist HYL1 in this process. (**d**) Base-to-loop processing. (**e**) Loop-to-base processing. The scheme presented in Figure 3 shows how complex metabolic machinery is involved in fine-tuning miRNA biogenesis when compared with the simplified picture presented in Figure 2.

Current knowledge defines several factors which have a positive or negative effect on RNAPII transcription of *MIR* genes. The first transcription factors described as proteins positively regulating transcription of *MIRs* were AT-NEGATIVE ON TATA LESS2 A and B (NOT2A/B), which interact with Carboxy-terminal domain (CTD) of RNAPII as well as DCL1 and SE [70]. The next discovered TF was the cell division cycle5 (CDC5) protein, which interacts with RNAPII, and also with DCL1 and SE. Moreover, CDC5 binds to promoter regions of *MIRs* and stimulates both transcription and pri-miRNA processing [71]. A multi-subunit complex—ELONGATOR—is another important element positively regulating transcription of *MIR*s, interacting with RNAPII as well as with microprocessor core proteins: DCL1, SE, and HYL1. Interestingly, ELONGATOR was described as a bridge-forming complex between chromatin and DCL1, providing a first strong suggestion that pri-miRNA processing may occur co-transcriptionally [72]. Another multi-subunit complex required for miRNA biogenesis is the MEDIATOR complex, which in general recruits RNAPII to *MIR* loci [73]. One of the latest reports showed a new player stimulating *MIR* transcription and interacting with MEDIATOR—the HASTY (HST) protein. It was believed that HST is required for miRNA export from the nucleus to the cytoplasm [74]. However, it has now become clear that its role is to recruit DCL1 to DNA, and to stabilize DCL1-MED37 (MEDIATOR subunit) interactions, which promotes transcription of *MIRs* as well as miRNA biogenesis (Figure 3b) [75]. Another complex—TRANSCRIPTION AND EXPORT COMPLEX2 (TREX-2)—was found to directly interact with DNA-directed RNA polymerase II subunit RPB1. TREX-2 also interacts with other factors involved in *MIRs* transcription, such as NOT2B, CDC5 and MEDIATOR. TREX-2 was found to be associated with microprocessor through direct interactions with SE and C-terminal domain PHOSPHATASE-LIKE1 phosphatase (CPL1). TREX-2 regulates phosphorylation status of HYL1 and recruits HYL1 and DCL1 to *MIR* loci [64]. Additionally, it has been shown that PRP40—an auxiliary U1 snRNP protein—also regulates co-transcriptional miRNA biogenesis. PRP40 interacts with the CTD of RNAPII and with SE. In the absence of PRP40, the association between DCL1 and chromatin is altered, which is accompanied by RNAPII accumulation at *MIR* genes. Retention of miRNA precursors at their transcription sites leads to a decreased amount of polyadenylated miRNA precursors without changing the amount of mature miRNA [76]. POWERDRESS (PWR) was found to promote transcription of *MIR172* genes by increasing RNAPII occupancy on their promoter regions. Moreover, such effect was observed only in in the case of three out of five *MIR172* genes (*MIR172a/b/c*) [77]. Growing evidence shows that *MIRs* transcription can also be regulated by miRNA-encoded peptides (miPEPs) [19]. In the case of 84 Arabidopsis pri-miRNAs, at least one open reading frame (ORF) was identified at the very 5′ end of the transcript. They are responsible for transcription enhancement of the pri-miRNAs from which they originate [43,78,79,80].

Several proteins/complexes are known to negatively affect general or selected *MIRs* transcription: suppressor of NPR1-1/CONSTITUTIVE1 (SNC1) as well as TOPLESS-RELATED PROTEIN1 (TPR1) form a heterodimer and high amount of each of these proteins in the nucleus leads to *MIRs* transcription downregulation [81]. POLYCOMB REPRESSIVE COMPLEX2 (PRC2) binds to *MIR156a/c* genes and introduces H327Kme3 mark that negatively affects transcription of these genes. Moreover, the level of H3K27me3 mark increases during plant development, decreasing the level of miRNA156a/c, which promotes vegetative-to-generative phase switch [13]. Another interesting example of *MIR161* and *MIR173* transcriptional repression was observed during salt stress: AGO1 binding to chromatin regions of these genes [82]. In plants, Ser7 residues from CTD repeats can be phosphorylated by CYCLIN-DEPENDENT KINASE F;1 (CDKF;1). It was shown that in the *cdkf* mutants, defects of pri-miRNAs 3′ end polyadenylation occur. Moreover, in *cdkf* plants, pri-miRNA and mature miRNA were decreased, but RNAPII occupancy across *MIR* loci was not affected [83]. These data indicate that CTD Ser7 phosphorylation is important for proper miRNA precursors processing.

From this short overview, a complex picture of *MIR* genes transcription emerges, showing that the interplay of various factors interacting with RNA PolII and microprocessor elements provides a sophisticated regulatory mechanism affecting the final microRNA level. All factors involved in regulation of *MIR* transcription are listed in Table 1.

In the same way as protein coding genes, *MIR* genes are transcribed by RNAPII and undergo the same regulation mechanisms through general transcription regulators. Several TFs have been identified which regulate transcription of selected *MIRs* genes. However, no general factor exclusively regulating all *MIRs* transcription has been found. Are there any features which distinguish *MIRs* loci from protein coding loci during the transcription process? This is one of the most interesting questions in this field. 

## 4. Splicing

Many plant miRNAs are encoded as independent transcriptional units. Plant *MIR* genes may contain introns. In this case, miRNAs can be encoded within exons (exonic miRNAs). MiRNAs can also be located within introns (in-miRNAs) of other protein-coding or long non-coding RNA (lncRNA) genes. The *MIR* gene structure is not conserved even for the members of the same *MIR* gene family within a given plant species. Within the Arabidopsis *MIR156* gene family, there are two genes transcribed as independent units: *MIR156a* and *MIR156c*, as well as two located in the other genes: miRNA156d, miRNA156f. (Supplementary Table S1, [89]). The Arabidopsis *MIR157* gene family is an example where all miRNAs are encoded by independent transcriptional units with *MIR157b*, *MIR157c* containing introns, while *MIR157a* is intron-less. (Supplementary Table S1). Pri-miRNA172b transcripts undergo alternative splicing (AS) and consequently, the mature miRNA sequence is located either in exon 1 (when intron is retained) or in exon 2 or 3 (intron excision, other splice isoforms) (Supplementary Table S1) [89].

Several reports of *MIR* genes containing introns are available for other plant species: for example, rice and barley *MIR444* family genes all contain multiple introns [90,91], as well as in the case of maize *MIR156c/b* [92] and barley *MIR166n* [93]. In rice, a total of 153 intronic miRNAs (in-miRNA), which is over 1/4 of the total rice miRNAs, were identified [90,94].

Both independent *MIR* genes as well as protein coding genes—containing miRNA— are transcribed by RNA Pol II. Transcripts, besides undergoing 5′ capping and polyadenylation, are subjected to constitutive or alternative splicing processes. Capping occurs co-transcriptionally and it is the first modification made on pre-mRNA/pri-miRNA. Firstly, 7mG is added to the first nucleotide on 5′ mRNA end just after synthesis of the first 25–30 nucleotides of transcript [95]. It was shown that the CAP-BINDING COMPLEX (composed of the CAP-BINDING PROTEIN20 (CBP20) and CBP80 proteins) affects proper processing of pri-miRNAs, including splicing. The Arabidopsis *MIR156a* and *MIR164a* genes contain introns. In *cbp20* and various *se* mutants, the abundance of these miRNAs decreases. This is accompanied by the accumulation of both spliced and unspliced pri-miRNAs, highlighting the role of splicing in efficient miRNA biogenesis [96]. These two *MIR* gene examples show a positive correlation between pri-microRNA processing and splicing efficiency.

There are several observations regarding the efficiency of miRNA biogenesis in the context of intron splicing:

(1) In Arabidopsis splicing and active 5′ splice site but not 3′ splice site, these are required for proper exonic miRNA163 biogenesis. Moreover, pri-miRNA splicing was affected by mutations in genes encoding important splicing proteins (the SR proteins (Serine and arginine-rich)); consequently, the miRNA level was decreased [97].
(2) Arabidopsis intronic pri-miRNA402 and mature miRNA402 accumulation was up-regulated by heat and correlated with splicing inhibition of the host intron-containing miRNA402 (AT1G77230, Supplementary Table S1) [98]. Heat stress activated the proximal intronic polyadenylation site downstream of the miRNA402 stem and loop structure and generated an intron-less, short pri-miRNA transcript.
(3) The RNA DEBRANCHING ENZYME1 (DBR1) is necessary for the regulation of genome-wide miRNA biogenesis in plants. Null *dbr1* mutants in both animals and plants are embryo lethal. In a weak Arabidopsis mutant allele of *DBR1*, *dbr1-2*, intron lariat forms accumulation was accompanied by a miRNA decrease in the case of miRNA156, miRNA159, and miRNA160, which are encoded by independent transcriptional units containing introns. It was found that two major microprocessor components, DCL1 and HYL1, were mis-localized in the *dbr1-2* mutant [99].
(4) In Arabidopsis, the splicing factor AAR2 (a homolog of U5 snRNP assembly factor in yeast and humans) associates with microprocessor proteins: DCL1, SE and HYL1. Interestingly, AAR2 is involved in HYL1 degradation in the cytoplasm and HYL1 dephosphorylation. In *aar2* mutant plants, the pri-microRNA level is reduced although the nonphosphorylated form of HYL1 (active in miRNA biogenesis) level is also reduced [100].
(5) Splicing of the barley transcripts containing intronic pri-miR160a and pri-miRNA5175a was induced by heat and correlated with the accumulation of mature miRNAs, suggesting the post-transcriptional regulation of miRNA precursor processing [101].
(6) In barley *MIR444c* gene, the sequences of miRNA444c*and miRNA444c are located in distinct exons separated by an intron. Only after proper intron removal can the pre-miRNA stem–loop structure be formed [91].

All these experiments show the profound effect of splicing on miRNA biogenesis. Splicing occurs co-transcriptionally and its flow may vary during various abiotic stresses, resulting in the regulation of miRNA biogenesis efficiency. Thus, splicing efficiency is another layer of miRNA regulation in response to environmental cues.

## 5. Pri-miRNA Processing

Plant pri-miRNAs are highly variable in length (from hundreds to thousands of nucleotides) and in secondary structure [102,103]. Their versatile characteristics put DCL1 in front of the difficult task of recognizing the proper position for cleavage. Correct pri-miRNA processing must rely on the structural features and accessory-guiding proteins, such as HYL1 and SE [56]. Plant miRNA precursors determinants for DCL1 cleavages are generally linked to the presence of short double-stranded segments of 15–17 bp, positioned below or above the miRNA/miRNA* duplexes [104,105,106]. Processing of the miRNA precursors is also favored by the presence of a G-C rich signature in the miRNA/miRNA* duplex region, emphasizing the importance of the secondary structure stability [107]. Additionally, there is a very strong preference against C-C and G-G mismatches in the DCL1 cutting position because these mismatches render the RNA region flexible, and they can affect the structural properties of the RNA secondary structure [107].

Another factor that has an influence on the stem–loop secondary structure is internal RNA modification. One of the most common mRNA and lncRNA (including pri-miRNA) modifications in plants is N^6^-methylation of adenosine (m6A) introduced by a writer mRNA ADENOSINE METHYLASE A (MTA; a homolog of human METHYLTRANSFERASE-LIKE PROTEIN 3 (METTL3)), the catalytic component of Arabidopsis m6A METHYLTRANSFERASE complex [108]. In Arabidopsis, in the absence of the m6A mark, pri-miRNA regions containing the miRNA/miRNA* duplexes are formed less frequently, which leads to a reduced level of 25% of all miRNAs [109]. The lack of m6A and consequent changes in the pri-miRNA structure could influence the binding of HYL1 during microprocessor assembly. On the other hand, MTA can directly interact with TOUGH (TGH) [109], an RNA-binding protein that binds to miRNA precursors. TGH can interact directly with HYL1 [110], contributing to HYL1–pri-miRNA interactions. Thus, the recognition of pri-miRNAs by HYL1 and the assembly of the microprocessor might be controlled by the presence of MTA, TGH and pri-miRNA m6A methylation. Hypo-N^6^-methylation could consequently lead to inefficient HYL1 and DCL1 recruitment to the miRNA precursors [109].

HYL1 is an important partner of DCL1, and its absence affects pri-miRNA cleavage accuracy. The lack of HYL1 results in strong developmental defects and low seed production caused by decreased levels of mature miRNAs belonging to at least 22 families [111], and the formation of abnormal miRNAs, which are derived from the regions beyond the stem of pre-miRNA [112]. Abnormal miRNAs originate mostly because of the incorrect selection of pri-miRNA cleavage sites. The proper function of HYL1 requires its homodimerization. It is proposed that HYL1 homodimers may ensure the distance from ssRNA-dsRNA junction in pri-miRNAs to direct DCL1 to cleave 15–17nt away from the junction [112]. However, there may be another explanation for the role of the HYL1 protein in miRNA biogenesis. The absence of the HYL1 protein might be bypassed by the lower temperature. Growing *hyl1-2* mutants at 16°C partially rescued both morphological and reproductive defects and restored some of the miRNA’s production to the wild type state [113]. The reason for this low temperature-dependent recovery is the increased structure stability of pri-miRNAs. The above results suggest that the binding of the homodimer of HYL1 may play a role in the stabilization of the proper secondary structure of some pri-miRNAs and, hence, the selection of the proper positions of the first cut by DCL1. Pri-miRNAs can also be stabilized using DAWDLE (DDL) and PLEIOTROPIC REGULATORY LOCUS1 (PRL1) proteins. Both can physically interact with RNA molecules and the DCL1 protein [114,115], positively influencing miRNA biogenesis (Figure 3c). On the other hand, factors such as CHROMATIN REMODELLING2 (CHR2), the ATPase subunit of the large THE SWITCH SUCROSE NON-FERMENTABLE (SWI/SNF) chromatin-remodeling complex and a partner of SE protein, can inhibit pri-miRNA processing. CHR2 acts as RNA helicase and can access and remodel pri-miRNAs’ secondary structure by disrupting the stem–loop structure bearing miRNA/miRNA* and reducing the mature miRNA level [86].

There are two different modes of pri-miRNA processing in plants. In the first one, called base-to-loop processing (BTL), the DCL1 cuts miRNA precursors at the base of the hairpin structure to release fold back pre-miRNA and then cuts again to generate mature miRNA in a manner that resembles DROSHA processing of pri-miRNAs in animals (Figure 3d) [102,116]. In the second one, called loop-to-base (LTB), the process has an inverted direction, starting from the terminal loop of the hairpin structure and proceeding toward the base (Figure 3e) [102,103]. Interestingly, in the case of long miRNA precursors with extensive complementary regions, independently of the localization of the first cut (BTL or LTB), processing always requires sequential steps of cleavages performed by the DCL1 for the proper release of mature miRNAs [102,103].

Lately, Gonzalo and colleagues have shown that the processing of pri-miRNAs occurs co-transcriptionally at pri-miRNA transcription sites as soon as the secondary structure of stem–loop is properly folded [117]. Their results have shown that there are differences in the processing of nascent BTL and LTBs pri-miRNAs. In the case of LTB pri-miRNAs, all miRNA biogenesis steps occur co-transcriptionally. Results for the BTL pri-miRNAs indicate that only the first cut occurs co-transcriptionally and that further processing of pre-miRNA takes place in the nucleoplasm. The authors have also shown that for some pri-miRNAs, both co-transcriptional and post-transcriptional processing co-exist and the ratio between co-transcriptional and post-transcriptional processing may vary depending on different environmental conditions. The dynamics of pri-miRNA processing may provide an additional regulatory layer for miRNA steady-state [117].

Interestingly, the processing of pri-miRNAs is promoted by the presence of DNA–RNA hybrid formation (R-loops) in the vicinity of the transcription start sites (TSSs). R-loops, especially in the antisense formation, have been identified as a new group of positive enhancers of miRNA biogenesis, promoting co-transcriptional processing of pri-miRNAs in plants. It is likely that co-transcriptional processing still occurs without R-loops but simply less efficiently [117]. Additional studies are necessary for the proper understanding of this process.

## 6. Pri-miRNA Degradation

There are three remnants of pri-miRNA particles after DCL1 cleavages that must be efficiently degraded: 5′ fragments of pri-miRNA, which starts from cap structure and end at the DCL1 cut; 3′ fragments of pri-miRNA, which start with DCL1 cut and end with poly(A) tail; byproducts of stem–loop structures above miRNA/miRNA* sequences.

**Figure 4:**
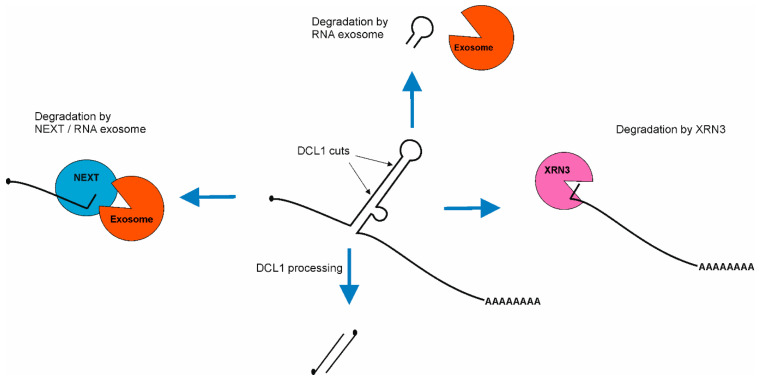
Scheme of pri-miRNA byproducts degradation after miRNA/miRNA* duplex excision. First, 5′ fragment of pri-miRNA is degraded using NEXT/RNA exosome machinery. Then, 3′ fragment of pri-miRNA is degraded using EXORIBONUCLEASE3 (XRN3). The apical fragment of stem–loop is degraded suing nuclear RNA exosome.

As such, 5′ fragments of pri-miRNAs are degraded by the nuclear RNA exosome machinery which starts the decay from their 3′ ends [118]. This process is supported by the NUCLEAR EXOSOME TARGETING (NEXT) complex, which targets these 5′ pri-miRNA fragments for degradation. The NEXT complex is recruited to pri-miRNAs through interaction with SE [119]. The degradation of 5′ fragments is very fast and efficient, as these byproducts are barely detectable in wild type plants but strongly accumulate in exosome mutants as well as in the NEXT complex mutants [118,119]. On the other hand, 3′ fragments of pri-miRNA are degraded using NUCLEAR EXORIBONUCLEASE3 (XRN3) [120]. Additionally, apical fragments of stem–loop structures are also degraded using the nuclear RNA exosome [118], but there is no information on whether the NEXT complex assists this process (Figure 4).

Recent discoveries have shown that both degradation machineries compete with the microprocessor for pri-miRNAs. First, genetic and molecular evidence revealed that the nuclear RNA exosome, together with its cofactor—the NEXT complex—degrades pri-miRNAs [119,121]. Two groups independently reported that plant crosses of microRNA biogenesis mutants (*se-2* or *hyl1-2*) with exosome-related mutants (*hen2* or *sop1*) lead to a partial restoration of the wild type phenotype. The level of both mature miRNA and miRNA targets is similar to those observed in wild type plants. However, the level of pri-miRNA in these double mutants is hundreds or thousands of times higher than in wild type [119,121]. Second, *xrn2* mutations restore the phenotype of another miRNA biogenesis mutant: *mac5* (MOS4-associated complex MAC5 subunit). This restoration includes miRNA as well as miRNA targets levels [122]. In exosome-related mutants, as well as *xrn*’*s* mutants pri-miRNAs, accumulation occurs, but miRNAs’ level remains unaffected in comparison to wild type plants. These findings lead to the conclusion that pri-miRNAs are produced in excess. Interestingly, both NEXT and XRN2 interact with SE and they are recruited by SE to pri-miRNAs. On the other hand, SE also interacts with many RNA binding proteins which are important for stem–loop structure stabilization (HYL1, TGH, MAC subunits: MAC3 and MAC5) [110,112,122,123] and destabilization (CHR2 and other RNA helicases: SMALL1 (SMA1), RNA HELICASE27 (RH27), RH6, RH8, RH12) [45,124,125]. Because of that, the current hypothesis indicates that pri-miRNAs in which stem–loop structure is properly folded and protected by RNA binding proteins are further processed, but pri-miRNAs without protected stem–loop structure are degraded. Moreover, experimental data indicate that SE plays a central role in defining/determining the fate of pri-miRNAs.

## 7. miRNA Stability

After the second cleavage with DCL1, the resulting miRNA/miRNA * duplex is prone to modifications at its 3′ ends. There are two modifications of miRNA/miRNA* duplex 3′ ends described in Arabidopsis, methylation and uridylation, which have opposite effects on RNA stability. The ribose of the 3′ terminal nucleotide of both strands is usually 2′-*O*-methyled through RNA methyltransferase HEN1 [126]. For its activity, HEN1 requires 2-nt overhangs within miRNA/miRNA* duplex and 2′OH and 3′OH of the 3′ terminal nucleotides [126]. The domains located in the N-terminal part of HEN1, two RNA binding domains—R1 and R2—and La-motif-containing domain—L—position RNA duplex in the C-terminal catalytic domain where 2′O-methylation occurs [61]. It was also shown that the 2′-*O*-methylation mechanism is Mg^2+^-dependent [127]. The lack of HEN1 results in the addition of one to five U residues at 3′ termini of miRNA [59,126]. In general, miRNA 3′ uridylation leads to 3′-5′ degradation of RNAs and is responsible for the reduced miRNA levels in *hen1* mutants [51]. The first identified miRNA TERMINAL URIDYLYL TRANSFERASE (TUTase) that adds uridyl nucleotides to miRNA 3′ ends in Arabidopsis is HEN1 SUPPRESSOR1 (HESO1) [128,129]. In the loss of function double mutant *heso1-1 x hen1-1/2*, the phenotypic changes and decreased miRNA accumulation, characteristic of *hen1* mutants, were partially rescued. Meanwhile, HESO1 overexpression in *hen1-2* mutants caused a further decrease in miRNA levels and a deterioration of the morphological defects [128,129]. Partial rescue of phenotype and miRNA levels in *hen-1/2 x heso1-1* plants prompted scientists to look for another miRNA uridylyltransferase. In 2015, Tu and colleagues showed that HESO1 paralog, UTP:RNA URIDYLYLTRANSFERASE1 (URT1), also affects miRNA uridylation [130]. However, UTR1 shows weaker enzyme activity in double mutants, lacking HEN1 and HESO1; the most abundant modification was single U added at the 3′ ends of RNAs. In addition, both enzymes show different preferences for nucleotides at the 3′ ends of miRNA: while HESO1 favors miRNAs ending with U, UTR1 prefers miRNA molecules ending in A. However, UTR1 and HESO1 are able to efficiently cooperate in the tailing of two miR158 forms, the full length miR158 and 1-nt truncated miR158. In this case, UTR1 adds 1-nt to the shorter form of miR158, making it the HESO1 substrate [130]. The partial rescue of *hen1* phenotype was also observed when a mutation in the gene encoding ATRIMMER2 (ATRM2) protein was introduced into the *hen1* background. ATRM2, a 3′ exoribonuclease, acts on an unmethylated and unuridylated subset of miRNA/miRNA* duplexes showing strong bias for miRNA* [131]. Another player in miRNA degradation is SMALL RNA DEGRADING NUCLEASE1 (SDN1), which is able to trim the methylated 3′ ends of single-stranded small RNAs bound by AGO1. Trimming results in RNA uridylation by HESO1 and eventually leads to the degradation of miRNA [132,133,134].

Stability of miRNAs is assured not only by 3′ ends 2′-*O*-methylation but also by its binding to AGO1. Strong hypomorphic mutants of *AGO1* show reduced a level of mature miRNAs, while in weak *ago1* alleles, protection of miRNAs from trimming or uridylation could be observed [66,135]. Slicing or recognition of the target mRNA using RISC triggers miRNA trimming or tailing, allowing for miRNA degradation and AGO1 release [135]. Moreover, extended interaction of RISC with target mRNA results in AGO1 and miRNA degradation [135,136]. It was shown that AGO1 directly interacts with HESO1, URT1 and ATRM2, further indicating the active role of AGO1 in miRNA turnover [131,137,138].

## 8. RISC Formation

The mechanism of miRNA loading onto AGO1 is not fully understood yet. It is known that CONSTITUTIVE ALTERATIONS IN THE SMALL RNAS PATHWAYS9 (CARP9) is involved in the proper forming of AGO1:miRNA complex [69]. The lack of CARP9 impairs HYL1-AGO1 interaction and causes accumulation of unbound-AGO1. The data presented by Tomassi and colleagues suggest that CARP9 is a factor that facilitates the formation of a complex consisting of HYL1, AGO1 and probably HEAT SHOCK PROTEIN90 (HSP90), and assists in miRNA-loading onto AGO1 [69]. HSP90 is a molecular chaperon important for plant RISC formation [65]. The assembling of the AGO1:miRNA complex is also supported by REDUCTION IN BLEACHED VEIN AREA (RBV), a WD40 repeats-containing protein that is conserved in plants [139]. In *rbv-1* plants, an association of miRNAs with AGO1 was decreased in comparison to wild type plants. Moreover, the lack of RBV caused a shift to higher molecular weights of the AGO1:miR159 complexes, suggesting that RBV facilitates the dissociation of a complex from other RISC formation factors. RBV is also present in high molecular weight AGO1:miRNA complexes [139]. In addition, RISC formation is promoted by an importin β protein, TRANSPORTIN1 (TRN1). Similarly to the *rbv-1* mutant, the lack of TRN1 causes the decrease in miRNA association to AGO1 [140]. These data indicate the role of RBV and TRN1 proteins in the formation of the mature miRISC complex.

It is also worth noting that different miRNAs have different abilities to bind to AGO1. This ability depends on miRNA structure and sequence and/or factors bound to pre-miRNA. The limiting factor for the loading seems to be the amount of available AGO1 in a cell [141].

## 9. miRNA Export

MiRNA export is a stage of maturing miRNA that was not broadly studied until recently. Previously, it was believed that HST is responsible for miRNA export to the cytoplasm [142]. The main reason for this assumption was that HST is a homolog of animal EXPORTIN5 (EXP5) which exports pre-miRNAs to the cytoplasm [143]. However, the published results did not support this assumption, as the nucleo-cytoplasmic distribution of miRNAs is not changed in *hst* mutants. Instead, the lack of HST results in global changes in miRNA level [74]. In 2018, Bologna and colleagues showed that a subset of miRNAs is bound to AGO1 in the nucleus and exported to the cytoplasm as an AGO1-miRNA complex via the CRM1/EXPO1 pathway [63]. AGO1 contains nuclear-localization (NLS) and nuclear-export (NES) signals that enable AGO1 to shuttle between the nucleus and cytoplasm. In Arabidopsis plants expressing *AGO1* with a mutation in NES, mature miRNAs were accumulated in the nucleus, showing the inability of a miRNAs majority to leave the nucleus without AGO1 [63]. Similar accumulation of miRNA in nucleus has been observed in plants with a mutation in the gene-encoding TREX-2 subunit ENHANCED ETHYLENE RESPONSE PROTEIN 5 (EER5, also known as THP1) and NUCLEOPORIN1 (NUP1). In both cases, changes in miRNA distribution were also accompanied by AGO1 accumulation in the nucleus. In addition, THP1 interacts with NUP1 and colocalizes with it at the nuclear envelope. It was shown that AGO1 is associated with NUP1 together with a number of nucleoporins, exportins and importins [64]. However, some of the miRNAs are detected in the cytoplasm in unbound form,, suggesting they are exported to the cytoplasm as methylated duplexes without AGO1 assistance [141]. Intriguingly, Gonzalo et al. suggest that most of the miRNAs unbound by AGO1 are produced in a co-transcriptional way, whereas miRNAs bound by AGO1 are mostly produced in the posttranscriptional manner (Figure 3d,e) [117].

## 10. Export of miRNAs out of the Plant Cell

Despite the presence of all components needed for posttranscriptional gene silencing (PTGS) in a single cell, miRNAs can be exported to manage long-distance regulation of gene expression. The mechanisms discriminating between two pools of miRNAs, maturating and acting in the same cell and exported out of the cell, are not defined. To date, only two proteins, HST and KATANIN 1 (KTN1), have been attributed as necessary for miRNA export outside the cell. BARELY ANY MERISTEM1 (BAM1) and BAM2 receptor-like protein kinases favor the process, while the AGO1 protein acts against the miRNA export to the phloem. The proteins and mechanisms necessary in the recipient tissues to uptake the phloem mobile miRNAs are not known [144,145].

The HST protein is localized at the periphery of the nucleus [146]. *HST* loss of function affects many aspects of Arabidopsis development. The phenotype of *hst-1* includes the shortened length of the vegetative phase and accelerated flower initiation, reduction in the root and leaves’ size, as well as leaves’ hyponasty. Such traits can be attributed to altered miRNA functions. Park and colleagues showed that *HST* mutation decreases the cellular level of some of the miRNAs, including miRNA156 [74]. This can be attributed to the recently recognized role of HST linking miRNA transcription and pri-miRNA processing [75]. HST was shown to be necessary for maintaining the cell-to-cell and cell-to-phloem export of artificial miRNA, amiRSUL, and non-cell autonomous miRNAs, such as miRNA160, miRNA165/166 and miRNA395. Interestingly, the process does not involve HST export out of the cell. In addition, the HST presence is necessary only in source tissues of the mobile miRNAs [145].

The AGO1 protein, as previously described, binds miRNAs in the nucleus. AGO1 and RISC complexes are not detected in the phloem; therefore, it is not expected that miRNAs can be exported in a complex with AGO1 [145,147,148]. In contrast, AGO1 binding of free cellular miRNA duplexes retains them inside the cell to downregulate target gene expression and to prevent further export [148,149]. Nevertheless, it was shown that a pool of 2′-*O*-methylated miRNAs escapes AGO1 binding [141]. Interestingly, some miRNAs have variable AGO1 loading efficiency between tissues [148,149]. This is simply explained by the tissue-specific differences between the expression level of the AGO1 protein. Consequently, AGO1 overexpression increases the level of AGO1-bound miRNAs. In the case of miRNA165/6, AGO1 binding of the cytoplasmic miRNAs is regulated by microtubules [150]. Microtubules suppress the cytoplasmic loading of AGO1 and promote the export of miRNA165/6. KTN1, a subunit of the microtubule-severing enzyme, is necessary for maintaining the mobility of miRNA165/6 and normal xylem patterning in roots. Consequently, KTN1 presence is indispensable only in cells exporting miRNAs, while its lack in miRNAs recipient cells does not diminish PTGS. Reduced mobility of miRNA165/166 in root stele and atypical xylem development is also observed in the absence of BAM1 and BAM2 receptor-like kinases. The BAM1 and BAM2 are plasma membrane and plasmodesmata localized, respectively, but their exact function in enhancing miRNA mobility is not known [144].

The presence of miRNAs in phloem sap suggests a physiological role in long-distance regulation of gene expression. During phosphate starvation, *MIR399* is highly expressed, and the mature miRNA399 is exported from shoots to roots to downregulate the target transcript *PHOSPHATE2* (PHO2, or UBC24 encoding the UBIQUITIN-CONJUGATING E2 enzyme) [151]. MiRNA156 is a phloem transmittable molecule responsible for tuber formation in potato (*Solanum tuberosum*) [152]. In potato plants grown in short days, miRNA156 migrates from shoots to stolons to favor tuberization. Overexpression of miRNA156 in potato leads to tubers formation in aerial parts of the plants. Such abnormal tuberization is also induced by a short day light regime [153]. Long distance trafficking of a substantial number of miRNAs across a plant organism was detected in soybean (*Glycine max*) and common bean (*Phaseolus vulgaris*) interspecies grafts [154]. Interestingly, miRNA’s movement is mostly unidirectional. The major flux of miRNAs is exported from shoot-to-root, a part of the graft or scion species. Additionally, the comparison of the low amount of root-generated miRNAs with the large number of shoot-to-root-transmitted miRNAs revealed a relatively low activity of miRNA biogenesis machinery in root tissues. The low production of miRNAs in roots makes the unidirectional shoot-to-root movement functionally essential and requires further studies [155]. Arabidopsis scion grafted on *Nicotiana benthamiana* rootstock also exports miRNAs to roots, while the reverse flux is minor. Interestingly, among the six *Nicotiana* root-to-Arabidopsis-shoot-transmitted miRNAs is miRNA156, responsible for the juvenile growth of plants [156].

Plasmodesmata connections between distinct plant species occur naturally during the interaction of parasitic *Cuscuta campestris* with its hosts. Whether the mobile miRNAs are transferred by the existing interspecies plasmodesmata connections was not studied. Nevertheless, the colonization of Arabidopsis by *C. campestris* stimulates the synthesis of *C. campestris* miRNAs specifically expressed in haustoria. The majority of the haustoria-specific miRNAs are 22 nt long and target Arabidopsis mRNAs to generate secondary siRNAs. Identified targets of the *C. campestris* miRNAs are Arabidopsis auxin receptors: TRANSPORT INHIBITOR RESPONSE1 (TIR1), AUXIN SIGNALING F-BOX2 (AFB2) and AFB3; a plasma-membrane-localized kinase, BOTRYTIS-INDUCED KINASE1 (BIK1), required for pathogen-induced and developmental signaling; a phloem protein, SIEVE ELEMENT OCCLUSION-RELATED1 (SEOR1), which reduces photosynthesis products loss from the phloem after injury; and a predicted transcriptional repressor required for the root formation, HEAT SHOCK FACTOR B4 (HSFB4). The described Cca-miRNAs/interspecies target modules are not specific only to Arabidopsis, which is explained by the fact that *C. campestris* has many host plant species among eudicots [157,158].

Studies on plant–fungi interactions revealed that miRNAs are capable of acting non-cell-autonomously and further regulate gene expression of non-source organisms. There are several examples of these interactions; they are bi-directional and involved miRNAs are grouped under a common name as cross-kingdom miRNAs [159]. A set of *B. cinerea* sRNAs were proven to target Arabidopsis MITOGEN-ACTIVATED PROTEIN KINASE1 (MAPK1) and MAPK2, enhancing disease susceptibility during infection (see Figure 1) [36]. In that case, plant AGO1 is utilized as an effector protein. Reversely, plants are able to decrease pathogen virulence through cross-kingdom export of miRNAs. Cotton upregulates the expression of miRNA159 and miRNA166 in response to *V. dahliae* infection [35]. The two miRNAs are exported to fungi hyphae to target mRNAs of ISOTRICHODERMIN C-15 HYDROXYLASE (HIC-15) and Ca^2+^-DEPENDENT CYSTEINE PROTEASE (CLP-1), respectively (Figure 1). These proteins are essential for different aspects of *V. dahliae* virulence. HIC-15 stimulates hyphae growth, while CLP-1 is necessary for microsclerotia formation, which are stored in soil and initiate disease spreading. Cross-kingdom miRNA-driven gene silencing also favors beneficial plant–fungi interactions. The ectomycorrhizal symbiosis between the fungus *Pisolithus microcarpus* and the tree *Eucalyptus grandis* is facilitated by Pmic_miR-8 exported to *E. grandis* roots. Pmic_miR-8 has no specific target in *P. microcarpus*, while in *E. grandis* the recognized targets are CC NUCLEOTIDE BINDING AND LEUCINE-RICH REPEAT DOMAIN IMMUNE RECEPTORS (CC-NLR). Therefore, the Pmic_miR-8 abolishes host immune system signaling to stabilize symbiosis [160].

## 11. Evolutionary Conservation of Plant microRNA Biogenesis Machinery

Phylogenetic analyses on the miRNA biogenesis machinery confirmed the presence of the majority of microprocessor proteins in representatives of all major green plant lineages, including green algae, bryophytes, ferns, and seed plants [41,161,162,163,164,165]. Currently living green plants (*Viridiplantae*) can be subdivided into two lineages: *Chlorophyta* (majority of the green algae), and *Streptophyta* (embryophytes and their closest algal relatives, a grade collectively known as streptophyte algae). According to data reported by Wang and colleagues, two evolutionary transitions in the streptophyte algae genomes were identified regarding both miRNA and siRNA pathways, during which the origin and diversification of several small RNA pathway-related genes occurred. During the first transition, the appearance of DCL-New, DCL1, AGO1/5/10, and AGO4/6/9 in the ancestor of *Klebsormidiophyceae* and all other streptophytes could be linked to responses to abiotic stress as well as to the evolution of multicellularity in streptophytes. During the second transition, the appearance of DCL2/3/4, and AGO2/3/7, as well as HYL1, in the last common ancestor of *Zygnematophyceae* and embryophytes suggests their possible contribution to pathogen defense and antibacterial immunity [164].

To better understand the conservation and divergence of proteins governing miRNA biogenesis across the plant kingdom, we took a closer look at the domain architecture of the three core proteins of miRNA biogenesis, DCL1, HYL1 and SE together with AGO1 throughout the main groups of green plants lineage, since the domain architecture is a more rigorous parameter than protein sequence similarity in classifying homologous proteins [166]. We used Arabidopsis and *Marchantia polymorpha* DCL1, HYL1, SE and AGO1 protein sequences as a query for the investigation of homologous proteins in the other plant species. The obtained results show that even in evolutionarily distant plants, all four proteins are highly architecturally conserved. Our investigation revealed that homologs of the HYL1 protein contain two DSRM (double-stranded RNA-binding motif) domains in all studied species (Supplementary File S1). The oldest HYL1 homolog in the plant kingdom, according to data published by Wang and colleagues, was found in the *Zygnematophyceae* representative, *Mesotaenium endlicherianum* (streptophyte algae), with 46% and 37% sequence identity to Arabidopsis and Marchantia HYL1 proteins, respectively. It should be emphasized that no homologs of this protein have been identified in any other representative of chlorophyta and streptophyte algae, which may suggest that HYL1 originated in the last common ancestor of *Zygnematophyceae* and embryophytes [164]. As plant and animal lineages separated during evolution roughly 1.6 billion years ago, it was hypothesized that their miRNA machinery evolved independently. Although an SE homolog, Ars2, is known in animals as a partner of the microprocessor and Dicer [167], no HYL1 homologs were found in bilaterian animals. Therefore, the report of the presence of HYL-1 Like proteins (Hyl1L) in Cnidaria and Porifera was quite remarkable [168]. However, unlike HYL1 in plants, the sea anemone HYL1-like protein stimulates just the second stage of miRNA biogenesis in generating mature miRNAs from intermediate pre-miRNAs. These results suggest that an HYL1-like protein was already present in the last common ancestor of plants and animals [169].

Similarly to HYL1 evolutionary conservation, we found that homologs of the SE protein contain conserved SERRATE_Ars2_N—ARS2 domain architecture, regardless of the taxonomic position of the plant studied. Phylogenetic studies published in recent years have shown that single copies of *SE* orthologs are present in all lineages of the plant and animal kingdoms but were not identified in prokaryotes, suggesting that the SE protein encoding gene emerged as a single copy in the last eucaryotic common ancestor (LECA) [161,163,164]. Interestingly, in representatives of some embryophytes, such as moss *Physcomitrium patens,* water lily *Nymphaea colorata* or parasitic dicot *Striga asiatica,* the SE gene underwent a duplication event. Additionally, in *Ginko biloba, Oryza sativa* or parasitic dicot *C. campestris*, multiple copies of *SE* gene have been identified (Supplementary File S1). Whether these duplication events have functional implications is a matter of further study.

**Figure 5:**
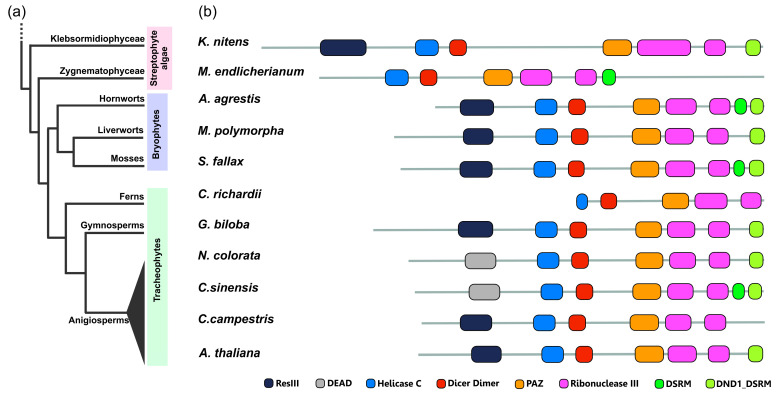
Conservation and divergence of DCL1 proteins across the plant kingdom. (**a**) Simplified phylogenetic tree representing relationships among major groups of the Streptophyta lineage. (**b**) Differences in domain architecture between DCL1 proteins from different representatives of plants: streptophyte algae—*Klebsormidium nitens* and *Mesotaenium endlicheranum*, bryophytes—*Anthoceros agrestis*, *Marchantia polymorpha* and *Sphagnum fallax*, fern—*Ceratopteris richardii*, gymnosperm—*Ginko biloba*, angiosperms—*Nymphaea colorata*, *Citrus sinensis*, *Cuscuta campestris* and *Arabidopsis thaliana*. Protein domains were annotated using the PfamScan tool and the Pfam database [153]. The figure includes the following Pfam accession numbers: DEAD (PF00270), Dicer_dimer (PF03368), DND1_DSRM (PF14709), DSRM (PF00035), Helicase C (PF00271), PAZ (PF02170), ResIII (PF04851), Ribonuclease III (PF00636).

Many of the key features of plant DCL1 important for proper miRNA processing are thought to have adjusted during the formation of evolutionarily advanced plants [170,171]. The distinctive organization structure of DCL proteins is thought to reflect their varied involvement in regulating gene expression. In our analysis, we found the greatest variation in the domain arrangement in the case of DCL1 proteins. For instance, the DCL protein from streptophyte algae *Klebsormidium nitens* resembles the same domain architecture as DCL1 proteins from *M. polymorpha* (Mp7g12090; MpDCL1b), *G. biloba* and *A. thaliana* (Figure 5). Our investigations revealed that most of the domain rearrangements in DCL1 orthologs across the green plants lineage concern N- or C-terminus. In most of the analyzed proteins at the very N-terminal part, either ResIII (Type III restriction enzyme, res subunit) or DEAD (DEAD-box helicase domain) domain is recognized. Both of them belong to the P-loop containing nucleoside triphosphate hydrolase superfamily (P-loop NTPases) and both display helicase activity. The presence of the ResIII domain in the DCL1 orthologs in the representatives of streptohyte algae and bryophytes and the appearance of DEAD domain only in angiosperms may indicate that the ResIII domain was an evolutionary archetype for the N-terminal part of the DCL1 protein. Additionally, in our analysis, we found some DCL1 protein examples lacking the ResIII/DEAD domain, such as in *M. endlicherianum* from Zygnematophyceae algae and fern *Ceratopteris richardii.* A characteristic feature of DCL1 proteins is the presence of one or two C-terminal double-stranded RNA binding domains (dsRBDs). There are two types of dsRBD domains recognized by the PfamScan tool [172] in the protein dataset analyzed in this study, a DSRM (double-stranded RNA-binding motif) domain and DND1_DSRM (double strand RNA binding domain from DEAD END PROTEIN1) domain. Both of these domains belong to the DSRM-like superfamily and, during green plant evolution, they are present in DCL proteins encoded in the streptophyte algae genomes with *M. endlicherianum* that possess the DSRM domain and *K. nitens* that possess DND1_DSRM (Figure 5). During the land plant evolution, most commonly, both dsRBDs domains or only DND1_DSRM characterize the C-terminal part of DCL1 proteins. Only in three DCL1 orthologs, from liverwort *M. polymorpha* (MpDCL1a), fern *C. richardtii* (Ceric.22G041000.1) and parasitic dicot *C. campestris* (VFQ95720.1), were none of these double strand RNA binding domains recognized (Figure 5 and Supplementary File S1).

The AGO protein family expanded during plant evolution with numerous duplications and losses [173]. Here, we focused on tracing the evolutionary and structural dynamics of AGO1 proteins in the green plant lineage. Similarly to HYL1 and SE proteins analyses, the domain architecture of studied AGO1 proteins is highly conserved. Three and two orthologs of the angiosperm AGO1/5/10 clade were identified in streptophyte algae *K. nitens* and *M. endlicherianum*, respectively, indicating that the ancestral form of this AGO protein clade originated long before plants’ terrestrialization. The domain composition of the streptophyte algae AGO proteins is identical to that identified in bryophytes representatives which lack a recognizable N-terminal glycine-rich domain that is present in Arabidopsis AGO1. From our analysis, it seems that this additional Gly-rich_Ago1 domain appeared after the core angiosperms diverged (Supplementary File S1). Recent studies on the origin and evolution of plant AGO proteins revealed that land plant’s AGOs are clearly distinct from those in animals and fungi, which suggests that the divergence of land plant AGOs occurred after the emergence of plants. Additionally, each of the AGO1/5/10, AGO4/6/8/9 and AGO2/3/7 clades included not only land plants but also streptophyte algae, which may suggest that the divergence of land plant AGOs may have occurred as early as in the common ancestor of streptophyte algae and embryophytes [162].

Altogether, our findings support the hypothesis that miRNA-mediated silencing machinery appeared at the early stages of plant evolution. We discovered that, in plants, the HYL1, SE and AGO1 proteins domain architecture is highly conserved. The lack of domain exchange or shuffling events suggests that these proteins evolved under strong control to maintain their long-term stability of biological structures. The most variation in the arrangement of domains occurs in DCL1 proteins. These data point out the divergent DCL protein regions which can be used for their functional characterization to investigate their impact on the miRNA biogenesis in the selected plant representatives. Due to these similarities and differences, many fundamental questions regarding core proteins of plants’ microprocessor remain unanswered. To determine the functional implications of these changes, further research is needed on these proteins.

## Figures and Tables

**Table 1:** List of known factors involved in the regulation of *MIR* genes transcription.

Factor	Full Name	Reference
Elongator	ELONGATOR complex	[72]
CDC5	CELL DIVISION CYCLE5	[71]
Mediator	MEDIATOR complex	[73]
HST	HASTY	[75]
NOT2	AT-NEGATIVE ON TATA LESS2	[70]
CPL1	C-TERMINAL DOMAIN PHOSPHATASE-LIKE1	[64]
STV1	SHORT VALVE1	[84]
PP4	PROTEIN PHOSPHATASE4	[85]
SMA1	SMALL1	[45]
CHR2	CHROMATIN REMODELLING FACTOR2	[86]
TREX2	TREX2 complex	[64]
PRP40	PRE-MRNA PROCESSING40	[76]
ILP1	INCREASED LEVEL OF POLYPLOIDY1-1D	[87]
NTR1	NTC-RELATED PROTEIN1	[87]
CDKF	CYCLIN-DEPENDENT KINASE F	[83]
SNC1	SUPPRESSOR OF NPR1-1, CONSTITUTIVE1	[81]
TPR1	TOPLESS-RELATED PROTEIN1	[81]
CDF2	CYCLING DOF TRANSCRIPTION FACTORS2	[88]

## Data Availability

Not applicable.

## Supplementary Materials

The following supporting information can be downloaded at: https://www.mdpi.com/article/10.3390/plants12020342/s1, Table S1: Structure of Arabidopsis *MIR* genes described in this paper; Supplementary File S1: Structure of microprocessor core proteins and AGO1 from representatives of different green plants divisions.
