# Supplementary material for: Recent Insights into Plant miRNA Biogenesis: Multiple Layers of miRNA Level Regulation
Source: Plants (Basel). 2023 Jan 11;12(2):342. doi: 10.3390/plants12020342 (PMC9864873; doi:10.3390/plants12020342)
Supplement: Supplementary file 1 [file plants-12-00342-s001.zip › Supplementary Table S1.pdf]

Supplementary Table S1. Structure of Arabidopsis *MIR* genes described in this paper. Presented data are derived from Arabidopsis *MIR* genes described in mirEX2 platform [89]. E – exon and I – intron following by the number of the exon or intron, respectively.

MIRNA	**miRNA Sequence**	**Independent transcriptional unit**	**Localization**	**Intron numbers**	**Locus/Host gene**
**miRNA156a**	UGACAGAAGAGAGUGAGCAC	Yes	E2	3	AT2G25095.1/-
**miRNA156c**	UGACAGAAGAGAGUGAGCAC	Yes	E1	2	AT4G31877.1/-
**miRNA156d**	UGACAGAAGAGAGUGAGCAC	No	I1	1	-/AT5G10946.1
**miRNA156f**	UGACAGAAGAGAGUGAGCAC	No	I1	2	-/AT5G26146.1
**miRNA157a**	UUGACAGAAGAUAGAGAGCAC	Yes	E1	0	AT1G66783.1/-
**miRNA157b**	UUGACAGAAGAUAGAGAGCAC	Yes	E1	1	AT1G66795.1/-
**miRNA157c**	UUGACAGAAGAUAGAGAGCAC	Yes	E1	1	AT3G18217.1/-
**miRNA158a**	UCCCAAAUGUAGACAAAGCA	Yes	E1	1	AT3G10745.1/-
**miRNA159a**	UUUGGAUUGAAGGGAGCUCUA	Yes	E1	0	AT1G73687.1/-
**miRNA159b**	UUUGGAUUGAAGGGAGCUCUU	Yes	E1	0	AT1G18075.4/-
**miRNA160a**	UGCCUGGCUCCCUGUAUGCCA	Yes	E1	1	AT2G39175.1/-
**miRNA160b**	UGCCUGGCUCCCUGUAUGCCA	Yes	E1	0	AT4G17788.1/-
**miRNA160c**	UGCCUGGCUCCCUGUAUGCCA	Yes	E1	0	AT5G46845.1/-
**miRNA161.1**	UGAAAGUGACUACAUCGGGGU	Yes	E1	1	AT1G48267.1/-
**miRNA161.2**	UCAAUGCAUUGAAAGUGACUA	Yes	E1	1	AT1G48267.1/-
**miRNA163**	UUGAAGAGGACUUGGAACUUCGAU	Yes	E1	1	AT1G66725.1/-
**miRNA164b**	UGGAGAAGCAGGGCACGUGCA	Yes	E1	2	AT5G01747.1/-
**miRNA164c**	UGGAGAAGCAGGGCACGUGCG	Yes	E1	0	AT5G27807.1/-
**miRNA166a**	UCGGACCAGGCUUCAUUCCCC	Yes	E1	1	AT2G46685.1/-
**miRNA166b**	UCGGACCAGGCUUCAUUCCCC	Yes	E1	2	AT3G61897.2/-
**miRNA172a**	AGAAUCUUGAUGAUGCUGCAU	Yes	E1	2	AT2G28056.1/-
**miRNA172b-5p**	GCAGCACCAUUAAGAUUCAC	Yes	E2	2	AT5G04275.1/-
**miRNA172e**	GGAAUCUUGAUGAUGCUGCAU	Yes	E1	0	AT5G59505.1/-
**miRNA173-5p**	UUCGCUUGCAGAGAGAAAUCAC	Yes	E1	0	AT3G23125.1/-
**miRNA319b**	UUGGACUGAAGGGAGCUCCCU	Yes	E1	0	AT5G41663.1/-
**miRNA395f**	CUGAAGUGUUUGGGGGGACUC	Yes	E1	0	AT1G69797.3/-
**miRNA399a**	UGCCAAAGGAGAUUUGCCCUG	Yes	E1	1	AT1G29265.2/-
**miRNA399b**	UGCCAAAGGAGAGUUGCCCUG	Yes	E1	1	AT1G63005.2/-
**miRNA399d**	UGCCAAAGGAGAUUUGCCCCG	Yes	E1	0	AT2G34202.1/-
**miRNA402**	UUCGAGGCCUAUUAAACCUCUG	No	I1	4	-/AT1G77230.1
